# Randomised controlled trial of an iPad based early intervention for autism: TOBY playpad study protocol

**DOI:** 10.1186/s12887-016-0704-9

**Published:** 2016-10-19

**Authors:** Joanna Granich, Alena Dass, Margherita Busacca, Dennis Moore, Angelika Anderson, Svetha Venkatesh, Thi Duong, Pratibha Vellanki, Amanda Richdale, David Trembath, Darin Cairns, Wendy Marshall, Tania Rodwell, Madeleine Rayner, Andrew J. O. Whitehouse

**Affiliations:** 1 Telethon Kids Institute, The University of Western Australia, Perth, Australia; 2 Krongold Centre, Faculty of Education, Monash University, Melbourne, Australia; 3 Centre for Pattern Recognition and Data Analytics (PRaDA), Deakin University, Geelong, Australia; 4 Olga Tennison Autism Research Centre, School of Psychology and Public Health, La Trobe University, Bundoora, Australia; 5 Menzies Health Institute Queensland, Griffith University, Gold Coast, Australia; 6 The Charles Street Clinic, North Perth, Australia; 7 Autism West, Mosman Park, Australia; 8 Telethon Kids Institute, 100 Roberts Rd, Subiaco, 6008 WA Australia

**Keywords:** TOBY, App, iPad, Intervention, Early therapy, Treatment, Autism spectrum disorder, Parents, Children, Technology

## Abstract

**Background:**

Evidence for early intensive behavioural interventions (EIBI) by therapists as an effective treatment for children with an Autism Spectrum Disorder (ASD) is growing. High-intensity and sustained delivery of quality EIBI is expensive. The TOBY (Therapy Outcomes by You) Playpad is an App-based platform delivering EIBI to facilitate learning for young children with ASD, while enabling parents to become co-therapists. Intervention targets include increasing joint attention, imitation and communication of children with ASD. The primary aim of the study presented in this protocol is to determine the effectiveness of the TOBY App in reducing ASD symptoms when used as a complement to conventional EIBI. The secondary aim is to examine parental attributes as a result of TOBY App use.

**Methods and design:**

Children aged less than 4;3 years diagnosed with ASD and parents will be recruited into this single-blind, randomised controlled trial using a pragmatic approach. Eligible participants will be randomised to the treatment group ‘TOBY therapy + therapy as usual’ or, the control group ‘therapy as usual’ for six months. The treatment will be provided by the TOBY App and parent where a combination of learning environments such as on-iPad child only (solo), partner (with parent) and off-iPad – Natural Environment (with parent) Tasks will be implemented. Parents in the treatment group will participate in a TOBY training workshop. Treatment fidelity will be monitored via an App-based reporting system and parent diaries. The primary outcome measure is the Autism Treatment Evaluation Checklist. The secondary outcome measures involve diagnostics, functional and developmental assessments, including parent questionnaires at baseline (T0), three months (T1) and six months (T2).

**Discussion:**

This trial will determine the effectiveness of the TOBY App as a therapeutic complement to other early interventions children with ASD receive. The trial will also determine the feasibility of a parent delivered early intervention using the iPad as an educational platform, and assess the impact of the TOBY App on parents’ self-efficacy and empowerment in an effort to reduce children’s ASD symptoms. The outcomes of this trial may have EIBI services implications for newly diagnosed children with ASD and parents.

**Trial registration:**

ACTRN12614000738628 retrospectively registered on 1^st^ of July, 2014. UTN: U1111-1158-6423.

## Background

The diagnosis of Autism Spectrum Disorder (ASD) in children is increasing globally [1–3], currently affecting approximately 1 % of the population [4–6]. ASD is characterised by social communication deficits and repetitive and restricted behaviours which place considerable psychological and financial pressures on primary care givers [7–10]. Early identification followed by clinical diagnosis of ASD typically occurs between the ages of 3 and 4 [11–13]. It is recommended that therapeutic intervention should soon follow diagnosis as early treatment has the ability to positively impact on young children’s neuroplasticity and thereby favourably alter developmental trajectories as evidenced in reports on the effectiveness of early intervention [14–18].

Empirical evidence for early intense behavioural intervention (EIBI) is now well established with significant and enduring impacts on the developmental and behavioural outcomes of young children with ASD [19–23]. A benchmark of EIBI of between 20 and 40 h per week for at least 2 years, targeting a range of pre-requisite developmental, adaptive and behavioural skills to harness optimal learning are recommended for children with ASD [15, 17, 24–26]. Early intervention services across many developed countries including Australia are unable to offer such intensive services to newly diagnosed children with ASD [27–29] due to shortfalls in trained staff and high costs associated with intensive therapy over an extended or indefinite periods of time [10]. Therefore, many families of young children with ASD are significantly constrained in their capacity to access EIBI. Long wait lists for early therapy services coupled with high ‘out of pocket’ costs limit families’ capacity to start and continue with early intervention. Since parents or primary care givers usually spend the greatest amount of time interacting with their children, they are in the best position to intervene and capitalise on their children’s neuroplasticity. Educating parents to become therapists for their children with ASD is not a new concept [30–35] and this approach may have lasting effects on early intervention outcomes [36–39]. Coaching parents to effectively engage, scaffold, prompt and reinforce communication and language can provide them with powerful skill-set for supporting their young children’s development and learning. This has been shown in studies focused on parent training of children with ASD using the principles of psychosocial and behavioural approaches [40–42] to facilitate behaviour change [40, 41, 43, 44] and to shift developmental trajectories [21, 45]. Parental involvement in early therapy has also been shown to reduce parental stress, enhance parental satisfaction and increase their confidence to harness opportunities for daily incidental learning for their children [46, 47].

Alongside EIBI and parent training, the proliferation of technology into the field of ASD treatment is growing. The development of touch screen tablets such as the iPad® has reimagined the portability, accessibility, affordability and social acceptance of obtaining information and education via this platform. Technology-based interventions and knowledge about autism treatment has seen some success in increasing communication skills among children with ASD by a way of augmentative alternative communication (AAC) or, on-screen social information [48–50]. Most recent report suggests that there are approximately 150 Apps that qualify for educational interventions using touch screen tablets [51]. As the technology market share and consumer base increases, new Apps are being developed and tested continuously. A systematic review of 15 studies focused on App-based interventions with individuals with ASD showed that the majority of these Apps were aimed at teaching academic, communication, leisure, activity transitions and employment skills [48]. However, few of these intervention Apps are underpinned by theoretical foundations about childhood development or provide evidence-based education for parents and the child with ASD in a variety of learning environments beyond conventional screen-based learning. Even fewer of these existing App-based interventions systematically monitor the child’s learning or provide feedback and guidance on the skills that the child is learning, or what the child should learn next. But, an intervention App that is consistent with key features of a comprehensive, individualised and quality EIBI is called the TOBY (Therapy Outcomes by You) Playpad App [52–54]. The TOBY App can be used by children or parents at any time solely, or in conjunction with other early intervention programs. Therefore, there is a potential saving of time and costs of the TOBY App over traditional EIBI that is typically expensive and delivered by a trained therapist coupled with parent education. A pilot evaluation of the TOBY App involving 33 families of children (age ≤ 16) with ASD were asked to use the App for six weeks without further instructions. This study showed high uptake of the TOBY App by parents with a consistent pattern of use throughout the TOBY curriculum [55]. However, lack of independent pre-post measures of children’s ASD characteristics and functioning as well as control group limited this study’s ability to determine change and evaluate the impact of the App on children’s development. Therefore, a large pragmatic trial is required to determine if the TOBY Playpad App can reduce core and non-core symptoms of ASD in newly diagnosed children in the community.

The objectives of this study are:

1. To determine the effectiveness of the TOBY Playpad App as a complement to early behavioural intervention as measured by a range of psychometric assessments which will assess core symptomology of ASD across time (baseline (Time 0), 3 months follow-up (Time 1) and 6 months follow-up (Time 2) through in-App data and parent reports.
2. To determine whether the response to TOBY varies with variation of treatment intensity across both, TOBY + ‘therapy as usual’, and across different TOBY learning environments and syllabus.
3. To measure the uptake of the TOBY intervention as recorded by the App and assessed by parents throughout the trial period using parent therapy diaries.
4. To examine attitudes, perceptions and beliefs of parents towards TOBY as an intervention method as assessed by specific TOBY, family and parenting questionnaires over 6 months.
5. To determine if TOBY intervention impacts on parental empowerment and stress levels.

We hypothesize that the TOBY App will improve children’s adaptive behaviours (language; social communication; restricted and repetitive behaviours) over time as assessed by a variety of measures at the final follow-up (6 months after starting the TOBY intervention). We also predict that parental empowerment in relation to the delivery of TOBY therapy will be enhanced at 6 months post randomisation. In addition, parental stress levels will be reduced as a result of the uptake of the TOBY intervention.

## Methods and design

### Participants

This study will include young children with ASD and their parents who will be recruited through state child development centres, diagnosticians, private service providers, parents’ support groups, community forums, local print media advertorials and public advertisements.

The eligibility criteria for the trial will include:

### Inclusion criteria

- *Children aged less than 4;3 months (51 months)*: At the time of screening for eligibility, potential participants’ age would be less than 4 years and 3 months. In Australia, the average age of a child with ASD at the time of formal diagnosis is 49 months [13]. The early key developmental areas that are primarily targeted by the TOBY intervention used in this trial calls for the inclusion of young children and their primary caregivers as necessary participants for this study.
- *Diagnosis of autism spectrum disorder:* Participants will be children who receive a clinical diagnosis of ASD according to DSM-IV-TR or DSM-V guidelines [56, 57] within the 12 months prior to the time of screening for eligibility. This post diagnostic time is a critical period for families affected by ASD where acceptance and management of the new developmental diagnosis is coupled with many decision making processes including which of a variety of disability agencies to access in a bid to seek funding, support and gauge treatment options. Families are typically wait listed for early intervention therapies that are supported by state jurisdictions, the Commonwealth of Australia or private therapy providers. The wait list period for ASD specific treatment services can vary greatly between states and regions for both, state or privately funded operators. For this trial, participants’ clinical diagnosis of ASD will be validated as part of baseline battery of assessments and will include children who also meet the diagnostic criteria for autism or autism spectrum on the Autism Diagnostic Observation Schedule – Generic (ADOS-G) [58] or the ADOS-2 algorithm [59] for DSM-IV/ICD-10 or DSM-V/ICD-11 [56, 57].

### Exclusion criteria

- *Previous use of the TOBY Playpad App.* Past exposure to the TOBY Playpad App will lead to contamination of the intervention and may contribute to a plausible spurious intervention effect.
- *Significant medical or sensory/motor impairments* such as a blindness or deafness which may incapacitate and thus impede the child’s ability to use a touchscreen device like the iPad®. This will also include unstable seizure disorder.
- *A traumatic head injury; genetic/neurological condition or disease* such as a diagnosis of Rett’s Disorder, Childhood Disintegrative Disorder or other neuro-developmental disorder of known aetiology such as Fragile X.

### Site recruitment

The TOBY Playpad trial will be conducted across two Australian sites: in Victoria (Monash, La Trobe and Deakin Universities) and in Western Australia (WA) at the Telethon Kids Institute. These sites have been selected based on the expertise of the multi-disciplinary research teams, the likelihood of meeting recruitment goals and the execution of the trial’s protocol as well as state specific funding. The primary coordinating site for this trial will be in WA whilst Victoria will be a satellite site with the main coordination occurring in Victoria at Monash University. Assistance with family recruitment and child assessments will be provided by La Trobe University whist technical support for the TOBY Playpad App across both sites will be provided by the computer science team at Deakin University.

### Study design

**Fig. 1:**
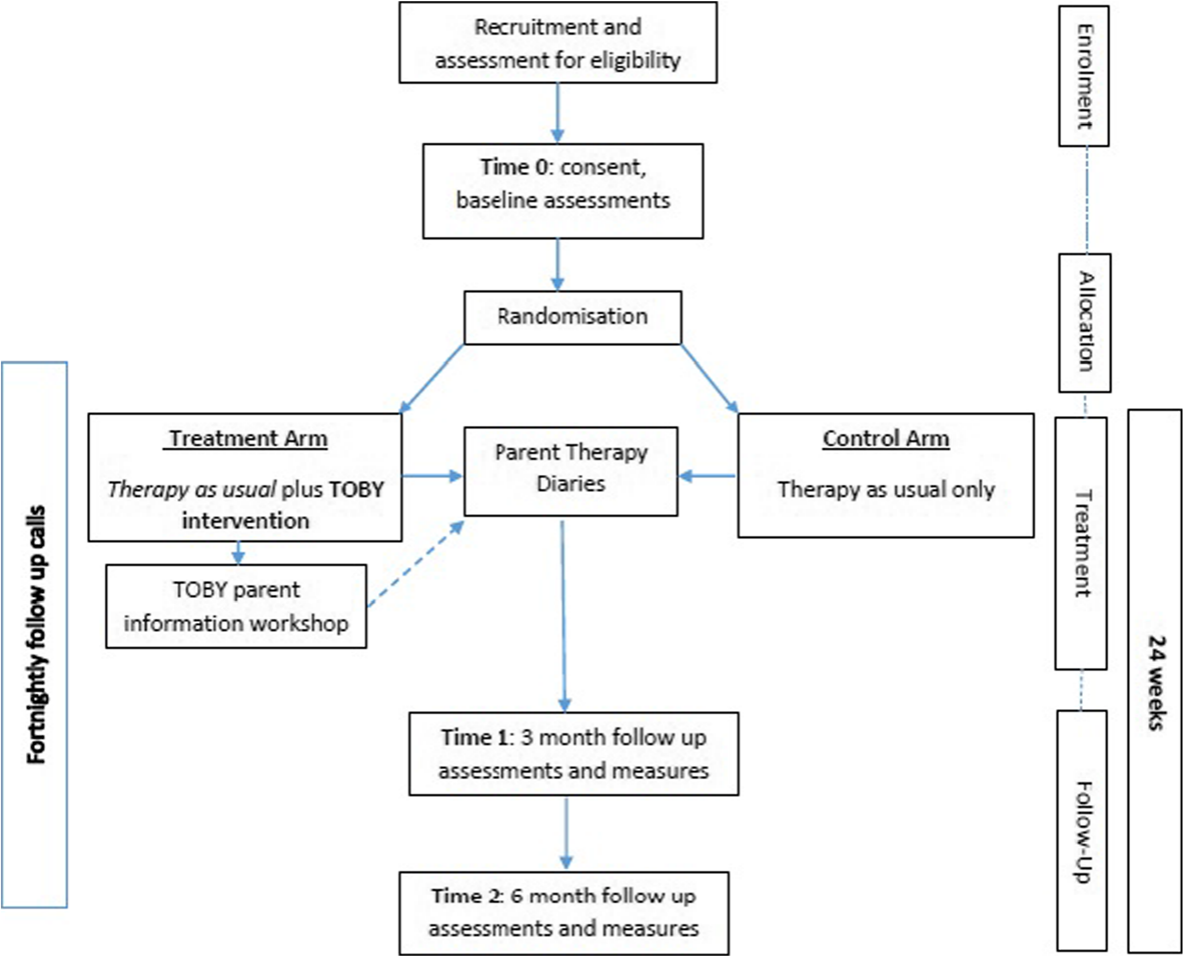
The CONSORT flow diagram for the TOBY Playpad trial

The TOBY Playpad study is a multisite, single (assessor) blind, randomised controlled pragmatic intervention trial with a parallel group design. Treatment in the form of early intervention delivered through an electronic platform like the iPad® using an application called the TOBY Playpad App in addition to ‘therapy as usual’ (TOBY therapy + therapy as usual) will constitute the treatment group. The ‘control’ group will receive any form of early intervention ‘therapy as usual’ accessed by the family through local service providers. Both groups will be assessed at baseline (Time 0) with two subsequent follow-ups at 3-(Time 1) and 6-months (Time 2) after the completion of baseline direct assessments including a battery of parent completed questionnaires. Figure 1 outlines the CONSORT flow diagram of this study.

## Trial status

This is an ‘ongoing’ study. The data collection has been completed and it is being prepared for analyses.

## Procedures

### Screening and enrolment

Referred families of recently diagnosed young children with ASD will be asked to contact the site specific clinical trial coordinator to obtain parent information form about the trial. Potential participants will be invited to engage in a brief (10 min) telephone screening interview which will be based on the eligibility criteria. This contact will provide potential families with the opportunity to ask questions about the trial. Upon meeting the eligibility criteria, parents and their child with ASD will be invited to enter the trial and undergo baseline testing (2–3 h) at respective trial sites. Written informed consent from parents of eligible children will be sought prior to baseline assessments.

### Baseline assessments, primary and secondary measures

A combination of direct observation and play-based assessments administered by a research -accredited ADOS assessor will be conducted on children with ASD. This will include parent self-reports on a range of child-related and ASD specific symptoms and behaviours. In addition, questionnaires about parental competencies, stress levels and services support will also be administered. The measures will be taken at trial time points outlined in Table 1.

**Table 1:** TOBY Playpad Trial measures

Measure	Time points T0, T1, T2^a^ unless otherwise noted	Description
Primary Measure Autism Treatment Evaluation Checklist (ATEC) [64]		The ATEC is a parent rated measure consisting of four subtest scales: Scale I. Speech/Language/Communication (14 items—with scores ranging from 0 to 28); Scale II. Sociability (20 items— with scores ranging from 0 to 40); Scale III. Sensory/Cognitive Awareness (18 items— with scores ranging from 0 to 36), and Scale IV. Health/Physical Behaviour (25 items-with scores ranging from 0 to 75). The four subscale scores can be used to calculate a total score (with total scores ranging from 0 to 180). The higher the subscale and total score, the more impaired the participant. The lower the subscale and total score, the less impaired the participant.
Secondary Measures Autism Diagnostic Observation Schedule-Generic (ADOS-G) [58]	T0, T3	The ADOS-G is a semi-structured standardised observational assessment that uses questions and activities to elicit the communicative, social, and repetitive behaviours relevant to ASD diagnosis. There are four modules which are age and language dependent. Therefore, this trial will use Module 1 (for pre-verbal children or those using single words/simple phrases (10 activities with 29 accompanying ratings) and Module 2 (children with flexible phrase speech (14 activities with 28 accompanying ratings). A standardised severity score is generated for respective modules. The lower the severity score, the less impaired the participant.
Mullen Scales of Early Learning (MSEL) [61]		The MSEL is a standardised developmental test for children (from birth to 68 months) which includes interactive tasks completed by the child to measure cognitive ability and motor development. The MSEL includes 124 items that measure five specific domains: 1) Gross Motor; 2) Fine Motor; 3) Visual Reception; 4) Expressive Language; and 5) Receptive Language. Four cognitive scales (Visual Reception, Fine Motor, Receptive Language, and Expressive Language) sum to represent an Early Learning Composite Score which measures overall cognitive functioning. A T-score, percentile, and age equivalent score can also be generated for each scale.
Symbolic Play Test (SPT) [66]		The SPT is a non-verbal standardised assessment of young children’s early concept formation and symbolisation. The SPT consists of four independent situations where the child is presented with sets of realistic representational toys and their spontaneous play is observed and recorded on a standardised checklist. The test is scored on the number of meaningful responses and connections made by the child when the objects are presented.
MacArthur-Bates Communicative Development Inventories (MCDI) [67]		The MCDI are parent report scales used to evaluate early language competence. There are two versions of the MCDI; 1) Words and Gestures (MCDI-WG) and 2) Words and Sentences (MCDI-WS). The MCDI-WG form is for 8–18 months old children while the MCDI-WS is for 16–30 months old toddlers. In both forms, parents report their child’s language development (i.e., vocabulary syntax). The MCDI were adapted for the Australian context with permission of the publishers, Paul H Brookes Publishing Co, (http://www.brookespublishing.com/customer-service/rights-permissions/)
Measure	Time points T0, T1, T2^a^ unless otherwise noted	Description
Communication and Symbolic Behaviour Scales - Developmental Profile (CSBS-DP) [68]		The CSBS-DP is a standardized assessment that measures communication and symbolic abilities as reported by a parent. The scale consists of a 24-item Infant-Toddler Checklist and assesses parents’ perceptions of their child’s communication and symbolic behaviour. The CSBS DP measures seven language predictors: Emotion and Eye Gaze, Communication, Gestures, Sounds, Words, Understanding, and Object Use. These predictors are summed to yield three Composite scores (Communication, Expressive Speech, and Symbolic). The composite scores are summed to yield a Total score.
Repetitive Behaviour Scale (RBS) [69]		The RBS is a parent questionnaire that characterizes the severity of repetitive behaviour. The RBS consists of 6 sub-domain scores: Stereotyped Behaviour (6 items - with scores ranging from 0 to 18), Self-injurious Behaviour (8 items - with scores ranging from 0 to 24), Compulsive Behaviour (8 items - with scores ranging from 0 to 24), Ritualistic Behaviour (6 items - with scores ranging from 0 to 18), Sameness Behaviour (11 items - with scores ranging from 0 to 33), and Restricted Behaviour (4 items - with scores ranging from 0 to 12). The six sub domains can be summed to yield a total score (total scores ranging from 0–129). Each item is rated on a four-point scale ranging from 0 (behaviour does not occur) to 3 (behaviour occurs and is a severe problem).
Family Empowerment Scale (FES) [70]		The FES is a 34-item rating scale assessing a family’s empowerment status, which is measured across two dimensions. The dimensions include the level of empowerment (family, service system, and community/political) and the ways empowerment is expressed (attitudes, knowledge, and behaviours). Each item is rated from on a 5-point Likert scale from 1 (not true at all) to 5 (very true). For example, parents will rate the following item: “I help other families get the services they need”.
Parenting Stress Index - Short Form (PSI-SF) [71]		The PSI-SF is a 36-item rating scale measuring the overall stress within the parent–child system. The PSI consists of three subscales: Parental Distress, Parent–child Dysfunction Interaction, and Difficult Child. Each subscale consists of 12 items rated from 1 (strongly agree) to 5 (strongly disagree), with subscale scores ranging from 12 to 60. The three domains combined form a Total Stress score (with a total score ranges from 36 to 180). A high score on the subscales and total stress score indicates increased levels of stress.
Parenting Sense of Competence Scale (PSCS) [72]		The PSCS is a 17-item scale that measures two dimensions of parenting self-esteem: Efficacy (scores range from 7 to 42) and Satisfaction (scores range from 9 to 54). A sample item includes “Being a good mother is a reward in itself”. Each item is rated on a 6-point scale from 1 (strongly agree) to 6 (strongly disagree). The total score of the 17-items represent parental confidence, with a higher score indicating a higher parenting sense of competency.
Measure	Time points T0, T1, T2^a^ unless otherwise noted	Description
Vineland Adaptive Behaviour Scale - Parent Version (VABS) [73]		The VABS is a standardized norm-referenced assessment tool used to measure adaptive behaviour. The Parent/Caregiver form includes 376-items. The VABS consists of five domains (each with 2–3 sub domains): Communication, Daily Living Skills, Socialization, Motor Skills, and Maladaptive Behaviour. An adaptive behaviour composite can also be calculated (with scores ranging from 20 to 160). Percentile ranks, adaptive levels, stanines, age equivalents, V-scale score, and standard scores can also be calculated from the raw scores
Behaviour Flexibility Rating Scale (BFRS) [74]		The BFRS is a 16-item scale assessing the behavioural flexibility in individuals with developmental disabilities. The BFRS consists of three subscales: Position/Location, Interruption/Disruption, and Interpersonal Mishaps. Using a 4-point scale, ranging from 0 (not a problem at all) to 3 (the situation causes severe problems); parents rate the severity of situations that can could be problematic to the individual. Higher total and subscale scores indicate greater behavioural inflexibility.
TOBY Playpad Intake Questionnaire	T0	The TOBY intake questionnaire consists of seven sections and is tailored to obtain parental report on children’s listening, communication, language and play skills. Items about children’s electronic media use and parents’ self-efficacy and confidence in assisting children to communicate are featured.
TOBY Playpad Follow-up & Satisfaction Questionnaire	T1, T2	The TOBY Playpad follow-up questionnaire version is as above. In addition, it contains 10 items and asks the treatment group parents (only) about the TOBY-App experience and satisfaction.
Case History Questionnaire (CHQ)	T0	The CHQ consists of eight sections and is tailored to gather socio-demographics of participants; birth; medical and developmental history of children with ASD. In addition, thinking styles of both biological parents in the form of Autism Quotient is asked.
TOBY therapy and therapy as usual Diary	T1, T2	Over 24 weeks of the intervention, both, intervention and control group parents report their children’s ‘therapy as usual’. In addition, the treatment group parents report TOBY App use termed as TOBY therapy. Both groups record relevant information at the end of each day in a diary format as featured in Fig. 3. Two diaries are provided to participants; 1) week 1–12 and 2) week 13–24.

^a^Data collection at T0 = baseline; T1 = follow-up 3 months; T2= follow-up 6 months

### Randomisation and blinding

Participants will be randomly assigned to either the ‘treatment’ or ‘control’ group based on stratification across three strata. The first stratum will be based on the trial sites (Telethon Kids Institute or Victoria). The second stratum will be Socio-Economic Status (SES) where SEIFA (Socio-Economic Indexes For Areas) for WA and Victoria [60] based on residential postcode will be used as a proxy for dichotomisation into two groups such as SEIFA decile 1 and 2 = Low SES and for decile 3–10 = High SES. The third stratum will be based on the developmental quotient (DQ) generated from the early learning composite score (cut point ≤ 50 DQ) on the Mullen Scale of Early Learning (MSEL) assessed as part of the baseline direct assessment battery [61]. A permuted block approach with a total of 8 strata (4 per site) will be used with random treatment allocation within each block of 20 randomisation sequences [62, 63]. The randomisation schedule will be generated by a computer and managed by clinical trial coordinator at the primary coordinating site in WA (JG). Clinical assessors and other investigators will be blind to the method of randomisation and treatment allocation. Given the nature of the treatment intervention, participating families are unable to be masked regarding treatment allocation. Each trial site coordinator will advise families of randomisation by telephone and a mailed letter.

### Sample size and analyses

The analyses for this trial will be performed on an intention-to-treat basis including all participants with outcomes data available. The primary outcome - a total score on the Autism Treatment Evaluation Checklist (ATEC) [64] (range 0–180) at Time Points 1, 2 and 3 will be analysed using a repeated measures ANOVA or via a General Linear Model (GLM). Based on the study investigator’s (AW and DC) clinical judgment in managing children with ASD, a score of 72 (50th centile) and a difference of 1SD = 32 (30th-70th centile) on the ATEC can represent clinically important improvement in ASD symptoms. The aim of this intervention is to look for a small-to-moderate effect size of 0.2–0.5 with 80 % power and two-sided alpha of 0.05. This will require a sample of approximately 64 participants per treatment group. Allowing for a 10 % drop-out rate, 70 participants will be required per treatment group, thus, the trial aims to recruit a total of up to 140 participants.

## Intervention

### Treatment group: TOBY therapy + therapy as usual group

This group of participants will receive treatment in the form of the TOBY Playpad intervention over 24 weeks, in addition to any other type of early intervention that the family and child are accessing upon entry to the trial. The TOBY Playpad intervention will be used solely or as a complement to ‘therapy as usual’. The TOBY Playpad (http://tobyplaypad.com/about/) is a unique early intervention program for children with ASD and other neurodevelopmental disorders. Currently, the TOBY Playpad is only accessible through an iOS-based device such as an iPod/iPad® App. The software, user interface, and theoretical underpinning of the TOBY syllabus including experimental evaluation of this assistive technology are described elsewhere [52, 53]. The TOBY (referred to from here) delivers a curriculum based on the principles of EIBI and evidence-based learning strategies. The TOBY curriculum contains a variety of user-generated and automated on-and off-iPad lessons. The TOBY is designed as an educational early intervention program for parents of newly diagnosed children with ASD and an automated (machine based) therapist for the child. The TOBY intervention is delivered in the following ways, through the availability of 30 on-iPad/on-screen and 300 off-iPad tasks. This can occur across three learning environments; 1) child only (solo), 2) parent and child (partner) or, 3) the Natural Environment Tasks (NET). The TOBY syllabus is designed to teach 52 foundation skills across four main learning areas (Sensory; Imitation; Language and Social) which are required for optimal language, communication, play and cognitive early childhood development. All TOBY lessons and tasks are developmentally sequenced to accelerate early learning and monitored by the App [52].

### Parent training

**Fig. 2:**
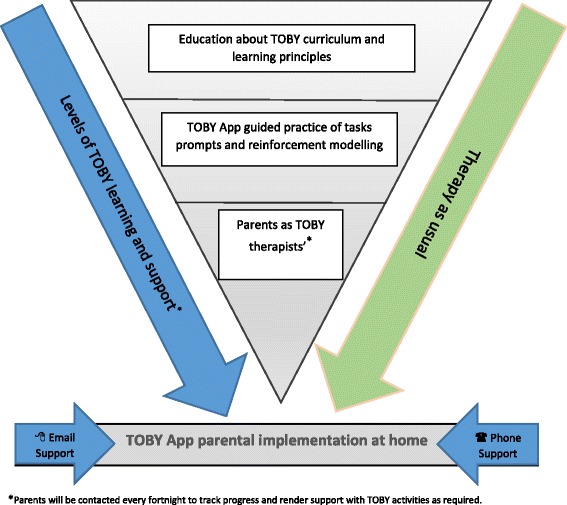
The parent workshop and training model for the intervention arm (TOBY therapy and therapy as usual) of the TOBY Playpad trial

One or both parents who will be randomised into the ‘*TOBY therapy + therapy as usual’* group will be required to attend a TOBY parent training workshop prior to starting the TOBY intervention. The parent training will be delivered using a combination of didactic teaching and parent coaching techniques about TOBY’s philosophy and its guiding principles. The overarching aim of this training is to empower parents to become therapists so that they can implement the TOBY curriculum over the next six months and beyond (see Fig. 2).

The parent workshop will be conducted by a TOBY consultant, who will be a trained therapist (speech pathologist or psychologist) with at least five years’ experience in working with children with ASD, together with the assistance of the trial coordinator at each site. This workshop will focus on providing parents with fundamental knowledge about TOBY. It will also equip parents with essential and practical skills on ways to implement and embed TOBY activities into daily interactions and play opportunities with their child. Parents will familiarise with the TOBY interface through in-App navigation across menu tabs, lesson plans and a variety of in-App guides. During this training session, parents will engage and practise with TOBY across several on-iPad and NET tasks, supported by the TOBY consultant. This informative hands-on session will provide parents with the capacity to start the TOBY intervention independently soon after the workshop. However, it will be recommended that parents allow up to 14 days post workshop to consolidate the newly learnt TOBY information, organise a ‘therapy tool box’ to support NET tasks and continue to learn and gain confidence about the TOBY intervention by using the App under a test flight mode. A parent user test flight mode will be created for each participating TOBY family. In addition, a TOBY intervention package will be given to families at the training workshop. This package will contain the following items; presentation slides and materials required for NET activities; TOBY essential and extended guides; iOS Guided Access; guide for connecting iPad to a Wi-Fi network; a loaned 16GB iPad and charger with a preinstalled version of the TOBY App (V1.4.1676); specific site iPad loan agreement forms; TOBY and ‘therapy as usual’ diary for weeks 1–12 (Phase 1); and a unique TOBY trial log-in registration user details in order to access the TOBY App including contact details for the site coordinator. This important information will be presented on a log-in card for reference and central storage in the family home. Furthermore, parents will be encouraged to use the provided hard copy TOBY guides in addition to accessing on-line resources through the TOBY website where instructional video’s demonstrating the implementation of tasks, various levels of prompting and reinforcements are provided (www.tobyplaypad.com).

**Fig. 3:**
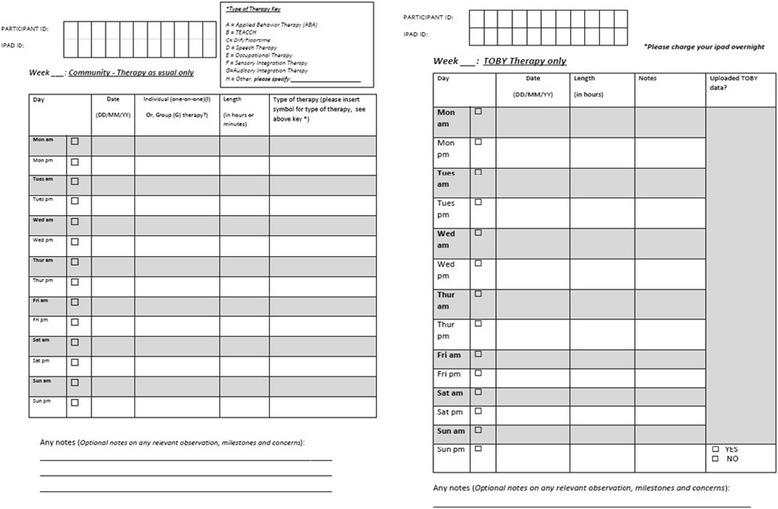
Parent report diary and log of TOBY therapy + therapy as usual

Parents will be recommended to start the TOBY intervention by firstly using the drag and drop tutorials with their child followed by the flexible range of on-iPad, partner and NET tasks on a daily basis for at least 20 min a day. Parents will be asked to upload their TOBY App progress to a secure and central TOBY server upon completion of tasks or at least fortnightly during the trial period. In addition, parents will be asked to complete a daily TOBY + ‘therapy as usual’ diary. In this document, parents will record the date; the amount of time spent in TOBY therapy plus other early intervention therapies, the format (individual or group based for therapy as usual) and the type of therapy the child participates in (see Fig. 3).

### Treatment fidelity

Monitoring of treatment fidelity is an essential component of any multi-site and complex intervention to ensure that the process of treatment evaluation is sound, valid and reproducible [65]. Parents will be asked to advise each study coordinator once they commence the TOBY intervention namely ‘TOBY start-up anniversary date’. From this date, families will be tracked during the intervention period. A scheduled fortnightly telephone call by the study coordinator to all intervention families will be made. This will take about 10–20 min depending on the primary care givers issues or concerns (if any). This will occur at least 10 times during the trial period (6 months). Prior to the scheduled contacts, each coordinator will monitor each participant’s progress with the TOBY intervention. This will be performed by accessing each participant’s TOBY user account via the TOBY Playpad website. This will assist the study coordinator to forge a transparent conversation with the primary care giver to ascertain parental status with implementing the TOBY intervention and to discuss concerns or difficulties that she/he might be experiencing. Access to the TOBY consultant will be offered to families if further support is required during the trial period that is beyond the coordinator’s capacity. All instances of support will be documented.

This process will assist with rapport with trial families and offer support in light of any troubleshooting required with the TOBY intervention or any technical problems with the App. The study coordinator will also remind parents to record all early intervention therapies (‘therapy as usual’) that their child is receiving in the provided diaries including time spent with the TOBY App across all three learning spaces (solo, partner or NET).

### Control group: therapy as usual

This group of children and parents will not receive the TOBY intervention. They will continue to receive their regular early intervention or may commence other type of early intervention therapy of their choice during the trial period. This group of participants will undergo the same number and order of direct assessments as the treatment group. They will also complete the same questionnaires and assessments as the TOBY therapy group at three and six months. The study coordinator will remind parents to record all early intervention therapies (‘therapy as usual’) that their child is receiving in the provided diaries.

### Follow-up 1 (T1) at 3 months

At 12 weeks, families in both groups will be asked to undergo their first follow-up assessments and return phase 1 therapy diaries. T1 will comprise two direct child assessments and a repeated battery of parent-reported questionnaires (see Table 1). A new, phase 2 (weeks13–24) therapy diary will be issued at this appointment. This testing session will take about 1.5 h. After that, fortnightly contacts with all families will recommence until the end of the trial.

### Follow-up 2 (T2) at 6 months

At 24 weeks, T2 will mark the end-point of the trial. Families’ will be invited to undergo a repeated battery of all questionnaires while the children will participate in three final direct assessments (see Table 1). This appointment will take approximately 2.5 h for each child. The TOBY therapy group will be asked to return the iPad and charger as well as completed Phase 2 parent diaries. All participants will be compensated for their time and effort in the trial by receiving an iTunes voucher which will enable them to access and upload the TOBY Playpad App onto their private iPad if they wish. At this point, the TOBY control group will be invited to receive a parent training workshop with the TOBY consultant including a TOBY intervention package for a period of 3 months. However, their TOBY App and therapy progress will not be monitored, recorded or analysed as part of this trial. Finally, we will thank all families for their participation in the trial and debrief.

### Data collection

The trial data will be captured on a source document named the TOBY Participant Report Form (TPRF), primary and secondary measurement forms. The TPRF will be a de-identified document containing each participants unique study number, screening information, a log of informed consent obtained, results of direct baseline assessments, randomisation, all fortnightly follow up logs during the trial, protocol deviations and follow-up assessment results across T1 and T2. A TOBY trial databases will be developed and all data entry will occur at each site. This pragmatic trial may render minor protocol deviations in terms of timing of the follow-up assessments. Therefore, a window of two weeks will be considered as an acceptable time frame for attending all follow-up direct assessments whilst the return of self-reported completed questionnaires within two-to-four weeks of each time point will be acceptable.

### Participant completion and withdrawal

Children in both arms of the study will be considered to have finished the trial upon completion of T2 direct assessments at 24 weeks post their baseline appointment. Those who do not to attend the 6 month follow-up direct assessments will be defined as withdrawn participants. This might reflect a parent or primary care giver’s decision to discontinue the TOBY intervention or to withdraw from the trial in general.

## Discussion

The primary aim of this trial is to determine the effectiveness of an iPad based EIBI, the TOBY App for improving behaviours associated with ASD in young children. The TOBY curriculum can be used as a complement to other early therapies. The TOBY can also act as an educational tool for parents by providing them with strategies to create regular incidental learning opportunities for their children on a daily basis. Overall, the TOBY has the potential to intensify the positive impact of any behavioural or developmental program for the treatment of ASD among young children. The results of this trial will provide evidence to assess the feasibility of parents becoming therapists for their child with ASD through a rigorous and yet pragmatic evaluation process. It will also provide evidence for the effectiveness of the TOBY App in improving trajectories for young children with ASD.

The strengths of this trial are inherent in its study design as a randomised controlled trial. Robust treatment fidelity measures as systematically gathered by the in-App reporting system together with parent-report diaries will enhance trial’s validity that may lead to future replication studies. This methodological characteristic will add rigour and value to this intervention trial as part of a quality assurance process that is ongoing throughout the study. Similarly, providing essential start-up TOBY parent-training and coaching together with scheduled monitoring of the TOBY intervention uptake will further demonstrate the essential fidelity features of research intervention trials. Collectively, this forms an important consideration when planning and executing randomised controlled trials of an intervention with a pragmatic approach.

The outcomes of this trial will contribute to evidence-based interventions designed to be used by parents in the community, soon after receiving a clinical diagnosis of ASD for their child. Further, the trial results will provide information about the usefulness of an App-based early intervention program at a critical point in time for many families who are seeking to start early intervention services but also who may be looking to empower and upskill their own understanding of ASD in order to reduce the impact of ASD symptoms on their child’s daily functioning, and to enhance their child’s capacity to learn.

Overall, the TOBY App is an innovative method of delivering EIBI as part of a home or community-based program. It is facilitated by technology and implemented by parents at a relatively low-cost compared to a qualified one-on-one therapist, or group early intervention. Research is needed to evaluate the effectiveness, feasibility and impact of the TOBY intervention in a rigorous clinical trial. The findings may have widespread implications for clinical and intervention service provisions for families of young children with ASD, thereby providing some relief to the stretched healthcare systems or education services for ASD, which generally are limited by the availability of public funds. The outcomes of this trial will also be widely disseminated through scientific conferences and seminars including parent networks and social media where the findings will create awareness about evidence-based ASD treatment which in turn should be used to inform end-user decisions for the uptake of early intervention as provided by the TOBY App.
